# Quality in general practice consultations; a qualitative study of the views of patients living in an area of high socio-economic deprivation in Scotland

**DOI:** 10.1186/1471-2296-8-22

**Published:** 2007-04-19

**Authors:** Stewart W Mercer, Peter G Cawston, Annemieke P Bikker

**Affiliations:** 1 General Practice and Primary Care, Division of Community-based Sciences, University of Glasgow, Glasgow, UK; 2 Drumchapel Health Centre, Glasgow, UK

## Abstract

**Background:**

Inequality in health and health care services is an important policy issue internationally as well as in the UK, and is closely linked to socio-economic deprivation, which in Scotland is concentrated in and around Glasgow. Patients views on primary care in deprived areas are not well documented. In the present study we explore the views of patients living in a high deprivation area on the quality of consultations in general practice.

**Methods:**

Qualitative focus group study set in an area of high socio-economic deprivation in a large peripheral housing estate in Glasgow, Scotland. 11 focus groups were conducted; 8 with local community groups and 3 with other local residents. In total 72 patients took part. Grounded theory was used to analyse the data.

**Results:**

Patients' perceptions of the quality of the consultation with GPs consisted of two broad, inter-relating themes; (1) the GPs' competence, and (2) the GPs empathy or ' caring'. Competence was often assumed but many factors coloured this assumption, in particular whether patients had experienced (directly or indirectly with a close family member) 'successful' outcomes with that doctor previously or not. 'Caring' related to patients feeling (a) listened to by the doctor and being able to talk; (b) valued as an individual by the doctor (c) that the doctor understood 'the bigger picture', and (d) the doctors' explanations were clear and understandable.

Relational continuity of care (being able to see the same GP and having a good relationship), and having sufficient time in the consultation were closely linked with perceptions of consultation quality.

**Conclusion:**

Patients from deprived areas want holistic GPs who understand the realities of life in such areas and whom they can trust as both competent and genuinely caring. Without this, they may judge doctors as socially distant and emotionally detached. Relational continuity, empathy and sufficient time in consultations are key factors in achieving this.

## Background

Quality in general practice and primary care can be conceptualised in a variety of ways, but a defining philosophy has been a holistic approach to care, and the centrality of the consultation and the doctor-patient relationship to this [1,2]. However, the recent quality agenda in general practice and primary care has focused more on access and bio-medical aspects of care than on relational aspects [3,4] and it has even been suggested that too strong a focus on the doctor-patient relationship may actually hinder quality improvement [5]. This philosophical 'drift' from the core importance of the consultation in general practice appears to be discordant with the views of 'rank and file' GPs; a recent survey of all GP Principals in Scotland showed continuing wide-spread support for holistic primary care, and frustration at a system which appears to discourage such a priority [6].

Inequality in health and in the provision and quality of health care services is a key policy issue internationally [7], in the UK as a whole [8], and in Scotland in particular [9,10]. Inequalities in health and health care are both closely linked to socio-economic deprivation [11], which in Scotland is concentrated in the west, especially in and around Glasgow [12]. In such areas of high deprivation, the concentration of health and social problems within families and the concentration of such families within practices result in levels of need and demand which place substantial and continuous pressures on primary health care teams. In such areas, primary care is often characterised by higher consultation rates, shorter consultation times and a larger list of problems to address within the consultation [13] and GPs report limiting influences of time [6] and stress [6,13].

Given the current political focus on inequalities in health care, there is a surprising lack of research on patients' views on the definition and determinants of consultation quality in the more deprived communities. In the present study we report these views, based on a qualitative investigation in an area of high deprivation in Glasgow.

## Methods

The analysis and results presented here are part of a wider action research project that aimed to improve the quality of healthcare services in a deprived area through participation with the local communities. Full details of the full study are being reported in a separate paper (Cawston P et al, submitted for publication). Ethical approval was obtained for the study from Greater Glasgow Primary Care Trust Research Ethics Committee and all participants gave their informed consent. The study was led by a part-time clinical academic (PC) as part of a masters degree in primary care. He was supervised by a senior non-clinical academic with expertise in qualitative research and focus groups. Four local residents were also an active part of the research team. The study was set in the locality served by a 'Local Health Care Co-operative', a small primary care organisation (PCO) in a peripheral housing estate in Glasgow, Scotland. The area covers a population of 22,700 people who are recorded as having high levels of socio-economic deprivation. The majority (53%) of people registered with the PCO are classified as being in the highest deprivation category in Scotland (DEPCAT 7) and all the practices within the local health centre are in the upper quintile of deprivation (based on mean patient deprivation score) within the health board area (Greater Glasgow and Clyde) (D.Mackay, University of Glasgow, personal communication). Standardised mortality in the area is considerably higher than the national average. PC also works as a part-time GP in the locality.

Data included in the present analysis was collected through eleven focus groups in which 72 people took part in total. The ages ranged from 16 to 80, and the majority (61%) of participants were women. In order to obtain a variety of perceptions and opinions on the topic, the participants were recruited from different community groups, including youth groups, groups for the elderly, men's groups, parents' groups, and other community groups, as shown in Table 1.

**Table 1:** Details of Focus Groups

List of Focus Groups Held	
Focus Groups	Number attending

Community Health Project (health volunteers)	7
Community group -people with disabilities	7
Community group – elderly	9
Mental Health community group	6
Family Learning Centre/Child Health/parent Group	6
Men's Group (alcohol problems)	9
Community centre (1)	6
Community centre (2)	4
Community centre (3)	5
Youth Group (younger teenagers)	5
Community Project (older teenagers)	8

Total	72

The 8 community groups were directly invited to participate in the focus groups and they invited members of their group to attend on an open basis (anyone who wanted to attend could do so). The 3 groups in the community centre (15 people in total) were recruited via a questionnaire in the local shopping centre and in the waiting rooms of the local health centre. Details of the questionnaire study (part of a the larger action research project) is being reported elsewhere (Cawston P et al, submitted for publication).

Eight focus groups were held with a variety of community groups and three in a community centre with local residents. The level of contact the participants had had with the primary health care services varied widely.

Focus groups are an established method for accessing personal experiences and encouraging people who feel they have little to say to formulate views through interaction with others. The aim of the focus groups was to permit a more in-depth exploration of issues and give those taking part a greater sense of personal contact with the project, possibly encouraging them to become personally involved with taking action over the subjects raised in the groups. The academic general practitioner and one of the local residents in the study group facilitated the focus groups together. Discussion was provoked by reference to a popular television medical drama. Five basic questions were used in all focus groups to provided a structure, but participants were encouraged to explore these and other issues that emerged in greater depth using personal experiences and by entering into discussion with others in the focus group. They were asked to evaluate their experiences, develop criticisms and explore what would be a good service.

(1) What's been a good experience of getting help with health problems?

(2) What's been a bad experience?

(3) What would you like to have been different?

(4) How do you think the health services which you receive could be improved?

(5) What sorts of things do you think would most improve the lives of people living in your local community?

The focus groups were audio recorded, transcribed verbatim. Computer software (NVIVO) was used in order to facilitate the analysis. All text was coded. However, for the purpose of this paper we report on views expressed about consultations with general practitioners (which formed a large part of the discussion in all groups).

### Analysis

A grounded theory approach was followed for the analysis [14]. Grounded theory is an emergent method, which seeks to find theory implicit in the data collected. Like action research itself, it seeks to understand a situation from 'the ground up' rather than starting with a pre-set hypothesis). Thus the initial data analysis of the participants' statements regarding the clinical encounter was inductive, and no categories were specified in advance. Based on careful reading and re-reading, the preliminary coding and categorization of the data was done independently by all three researchers on different focus group transcripts. Ideas about emergent themes were also recorded by all three independently ('memoing'). The extent to which the categories and emergent theories identified by the researchers corresponded was discussed and the emerging codes and theories were repeatedly discussed and refined through regular weekly meetings of the three researchers over a period of 3 months. The process of this utilised the constant comparative method – initially comparing data sets between focus groups, later comparing data with emergent theories. Issues were framed or given greater complexity to take into account contrasting views developed in the focus groups, thus providing a 'third order' analysis of the findings.

The second part of the analysis was more deductive, guided by the emergence during the first part of the analysis of two key factors (continuity of care and length of consultations) which appeared to be closely linked which the major themes identified regarding views on consultation quality. Thus our aim was to test the emergent theoretical proposition that these two factors in particular were direct importance to the core themes around quality of consultation. The interactions between (a) the categories concerning patients views on quality in the consultation and (b) the 'structural' factors of time and continuity of care was then further examined in two ways. Firstly, with the help of NVIVO's search option, we assessed the data for overlaps between each of the categories of the encounter and each of the two structural factors (that is, we searched for examples of when data had been double or triple-coded) comparing this with a third factor (access) which had emerged as a category, but did not appear to be closely linked to views on the consultation itself. In order to take into account the wider context in which the categories were grounded, we also took a proximity search that included the preceding and following five lines of the core codes about consultation quality. Finally, all transcripts were re-read in full order to check that all interactions were included (i.e., we thoroughly searched the data again for evidence that would confirm or disconfirm the emerging theories. Throughout the study, we used path diagrams in order to visualise and develop the relationships between the emerging categories and emerging hypothesis.

## Results

Two core, inter-related themes emerged regarding patients' views of quality of consultation with GPs. These concerned perceptions of (1) the perceived competence of the doctor, and (2) the doctors' empathic concern.

### Competence

This theme included references to the doctor's technical competence, including medical skills, knowledge, and training. However, perceptions of competence were also influenced by the patients' 'image' of the doctor as an individual, including appearance and mannerisms. In general, patients assumed that doctors were technically competent, seeing them as 'medical experts'. This was especially the case among the elderly patient group;

"*you say well he must know best, so everybody takes it that he does know best" (focus group B)*.

### Treatment and health outcomes

Patients' confidence in the doctor's competence was affected by prior experiences (or the experiences of other family members) with that doctor, and the effect they felt this had on their health outcomes and/or the treatment they received. Their perception of the doctors' competence was thus heavily reinforced by successful outcomes in the past;

*"I'll give you a classic example. I had bother with my bowels and they're back to normal now, and that's thanks to the doctor, who came and gave me medicines and that" (both focus group C)*.

*"...luckily enough the doctor came out and spotted this and got him emergency into the hospital straight away, and my dad used to say, thank you, thank you, you saved my life, if it wasn't for you I'd probably be dead, so I mean she is good" focus group F)*.

Conversely, the doctor's competence would be questioned when experiencing an apparently inaccurate diagnosis, lack of examination, and/or treatments which did not result in improved heath outcomes. In these instances patients would often be critical, more specifically placing judgements on the doctor's medical skills, knowledge, education, appearance or mannerism;

*"The doctor is just like a wee boy out of college, he was just a daft wee boy, and then the guy was like oh aye your chest is bad, my mum ended up having to get an ambulance and going up to the hospital" (focus group F)*.

*"Aye, I went down once and I had pure bellyaches and all that, and I went down and they said it was just a bug. And then two weeks later I had to the doctor out and got took into hospital. My appendix burst and I was in hospital for two weeks.... that could have been prevented if they examined us better" (focus group L)*.

### Patients' input on understanding of symptoms

At other times the competence of the doctor was judged on how seriously he or she took patients' own understanding of their symptoms, which were based on having had similar health complaints in the past or in relation to their children:

*"...I say I mean if you're there with that kid all the time you know that that's not normal for that kid so it might just be a wee silly symptom that you're seeing but you know that's not them so if you go to see a doctor they should take that on board that you know you might not be trained as a doctor but you do know your own kids" (focus group A)*.

Clearly there is considerable overlap between views on competence and views on the doctors' empathic concern and caring, which are further elucidated below.

### Empathic concern

Patients emphasised the importance of 'genuine' relationships with GPs. Patients perceptions of the 'approachability' of individual GPs had a powerful influence on the ease with which they felt they could talk to them. Being friendly and approachable however did not mean avoiding straight talking, which was seen as an important part of being "genuine". This was closely related to the idea of a "positive attitude" in which the doctor contributed energy, enthusiasm and a clear direction to a situation which otherwise appeared bleak and hopeless. Whether plain speaking was interpreted positively however depended on the context in which it took place. Without 'respect' – the direct approach simply belittled the patient.

Specific attributes involved (a) feeling listened to by the doctor and feeling able to talk; (b) feeling cared for and valued as an individual (c) feeling the doctor understood the 'bigger picture' (d) having, and being able to understand, explanations. These are further described below;

### Being listened to/being able to talk

As indicated above, patients described the ability to talk to the doctor and explain their health concerns as being dependent on the doctors 'approachability' – whether or not they felt the doctor was able and willing to take the time to listen and really pay attention;

*"I've got Dr X and Dr Y and the two of them have got totally different attitudes when it comes to talking to you, Dr X will take the time and listen to you... (focus group D)*."

### Being treated as an individual

This theme related particularly to the feeling of being respected as an individual as opposed to being 'treated as a number':

"And going away they forget about you most of the time I think as soon as you walk out of that door" (focus group A)

*"your body's just a machine to them"(focus group B)*.

This also related to perceptions of the doctors' sensitivity (or lack of sensitivity) towards the patient, and non-judgmental (or prejudicial) attitude;

"*When I go in to see my doctor, I want him to see me, the person, not a bottle of methadone, I'm not that, I'm a person that's got needs and everything like every body else, because I'm on methadone, I just don't get treated properly (focus group I)*

### Understanding the 'bigger picture'

This theme related to a strong desire by patients to tell their 'story' in the consultation, and to feel that the GP genuinely understood the 'bigger picture' in relation to their wider environment, such as family issues, poverty, and community problems.

Many patients felt that because the GPs did not live or socialise in the area, they could therefore not really understand "the sort of life that people in Drumchapel are actually living". For example, advice on 'healthy living' was often regarded as failing to adequately take into account the realities of daily life in the area. The many examples given of 'social distance' included GPs lack of awareness of limited local shopping facilities, public transport issues, difficult choices on low incomes, and the effects of poor housing, conflicting family demands, fear of violence, and social isolation.

#### Having explanations/being able to understand

This theme referred to explanations and information given by GPs. Patients described the need for more explanation in a way they could understand. Patients often felt that GPs *"just prescribe" *without clear explanations of how to use the medication, the potential side effects, or why a certain treatment is given. Alternative ways of gaining such information included going to see the practice nurse in the health centre, attending a support groups, or looking up medical books. In all groups there was a desire for more information, but this did not necessarily reflect a desire for participation in decision making. Younger patients, such as parents of children with medical problems were more eager to participate in 'shared-decision making'. However, in general it was a lack of clear explanations that resulted in confusion and stress, whereas understandable explanations could result in better concordance;

*"...I'm kind of afraid to take tablets and I not one of these, you know a pill taker, you know if I have to take them, but I remember that once she explained what these tablets would do and that you have to take them, you don't want to take a stroke..." (focus group B)*.

*"... some of them make you feel inadequate, you know you're getting old and you're getting senile and you're not able to take things in, I mean sometimes you feel that way, you feel as if och I'll just lift my bag and jacket and go" (focus group B)*.

## 'Structural' factors – continuity, consultation length, and access

Consultation length and continuity of care emerged as the two key 'structural' factors that were intimately associated with patients accounts of quality reported above. Both influenced and overlapped with views on competence and on caring in all focus groups.

### Continuity

Seeing the same doctor over time was generally seen as the basis of the 'genuine relationship' which patients sought. It enabled them to be able to express their concerns, feel valued, and trust the doctor's expertise, because of the doctor's knowledge, awareness and understanding of their condition and situation. However, simply seeing the same doctor over time was not in itself a guarantee of such a therapeutic relationship. Such temporal continuity was seen in negative terms if the GP was felt not to genuinely engage with patients and their problems. For some of the younger patients, the idea of seeing the same doctor too frequently fed beliefs that the doctor could becomes complacent or 'bored' with their problems, resulting in missed diagnosis or simply feeling that their concerns were no longer being taken seriously.

*"it isn't like they take the time and listen to what you say, it's just like, he's here again the same complaint, get him out the door" (focus group F)*.

By seeing different doctors, such patients felt they were in a better the position to compare doctors against each other and thus reach a judgement about quality of care. However, patients who knew their doctor well, and had a good relationship with their doctor (more often but not exclusively those with chronic conditions) deeply valued this structure, and were unhappy about having to see different doctor, because of problems in the availability of their 'own' doctor.

### Time available in the consultation

The theme of the doctor 'taking time' (or perhaps more accurately 'giving' time) was a strong one and related to both views on competence and on caring; due to the perception of time being 'precious' and the doctor being a 'busy person', experiences of the doctor 'taking the time' led to feelings of being valued as an individual and care about. Lack of time was frequently seen as a major limitation on the quality of the consultation. Moreover, with consultations being perceived as 'rushed', the time pressure added to feelings of stress in patients in the sense of taking up the doctor's valuable time, not having time to express all concerns, or even forgetting some of the reasons for attending;

*"The present doctor's system you're in the door and out of the door as quick as possible*... *the doctor's at the door before you've even got your jacket on... and you're "I forgot about that" but you're out the door." (focus group A)*

"your just a number and you just go in and go back out and that's it, if I've ever been in a doctor's surgery any more than 5 minutes I'm like totally shocked and that includes for me and the children, know what I mean I just feel as if you get shoved in and shoved back out again." (focus group F)

"You feel as if you're taking up his time...that makes you feel under pressure" (focus group B);

*"...at your own GP there's... the file... before you're... and you're "I forgot about that" but you're out the door" (focus group A)*.

However, one person felt that out that spending 'too much time' with the doctor could be also be anxiety provoking, perceiving this as meaning that the doctor thought there was something "seriously wrong".

"Doctors always seem to be under pressure for time to get as many patients in as possible. The average consultation down at the health centre in my experience is five, seven minutes and that's a long time. They all know if you're in longer than that then it's something serious you know." (focus group B)

### Access

The different ways within which an appointment was obtained was discussed across the focus groups, for example, how the number of days waiting for an appointment varied due to the different systems used in different health centres. However, no clear theoretical link was found between waiting time to get an appointment and the experience of the clinical encounter itself. In contrast to continuity and consultation length, there was little overlap between access and consultation quality themes in the narratives and discussion sequences in the transcripts of the focus groups. This does not mean that access was not an important issue to patients – rather it was not conceptually linked to the consultation itself, which was the focus of the present analysis.

Waiting for an appointment to see the doctor, as well as waiting in the waiting room were often simply seen as being part of the experience of going to the doctor. For a few participants, waiting a long time in the waiting room (if the doctor was running late) influenced the consultation in a negative way by the build up of stress and anxiety (e.g., feeling uncomfortable in a crowded space, being seen by others, being 'publically' asked personal questions by the receptionists, or being a parents and trying to keep children under control.

## Discussion and conclusion

The present study has shown that patients living in a high deprivation area in the UK view quality of consultation in general practice as relating both the GPs' competence, and the GPs empathic caring. Knowing a specific GP and being able to see that GP, and the amount of time available in the consultation were recurrent issues that were deeply intertwined with the two key themes of competence and caring.

### Strengths and weaknesses

A key strength of the present study was that it was carried out in an area of extreme socio-economic deprivation, and as such represents one of very few qualitative studies done in such a setting. It took a community approach to sampling, and thus the voices of a diverse range of community members were heard, including 'hard to reach' groups such as young men. It was a large sample for a qualitative study, and we are confident that 'saturation' was reached in terms of themes and viewpoints.

An obvious weakness was that the study did not include the 'opposite' socio-economic group, i.e., patients from an affluent area, and thus a comparative analysis was not possible. Secondly, the choice of focus groups as the enquiry arena may have inhibited some individual participants from voicing issues or concerns that may have become apparent in individual one to one interviews. Thirdly, only one high deprivation area in Glasgow was sampled. The views of patients of different ethnic origins were not included, as all participants were white Caucasians. Fourthly, the focus groups were conducted by PC, a part-tem academic GP who works in the locality. Clearly this may have influenced what patients felt they could say and how they stated their views, but we were aware of this possibility throughout the project, and in the analysis and took a reflective approach with this in mind. One of the researchers, AB, had no involvement in the project prior to the analysis, and as a non-clinician with experience in qualitative methods her contribution to the analysis and the discussions concerning the analysis also helped to balance ant pre-conceptions held by PC and SM, both academic GPs.

### Relationship with other studies

#### Deprivation and consultation quality

As far as we are aware, the present study is the first qualitative study of the views of patients living in a deprived area on consultation quality in general practice in the UK. However, two qualitative studies from the USA support the findings in the present study. In the first, trust in physicians by low-income female patients was found to be strongly related to continuity, communication, and perceptions of caring, and competence [15]. Although we did not explicitly explore the theme of trust, the parallels with the above study in terms of the emergent themes are striking. The second, a study of 60 African Americans with chronic illness [16], found greater dissatisfaction with health care amongst low-income patients compare to middle-income patients, with reports of feeling not listened to, being treated in a condescending manner, and concerns being 'brushed off by physicians, and consequently they questioned the physicians competence and knowledge.

In a quantitative study, socio-economic status differences between African Americans and healthcare providers accounted for differences in patients' perceptions of respect by physicians, and patient satisfaction [17]. The author attributed part of this to a concept of 'social distance' between provider and patient. Interestingly, in the present study similar views on social distance were expressed, with several patients remarking that GPs, although working in the community, didn't live in the community and therefore often couldn't understand the realities of life in such a high deprivation area. GPs who provided sufficient time in consultations, were genuinely empathic, and provided relational continuity were to some extent 'exempt' from this judgement of social distance, and were more likely to be regarded as understanding the 'bigger picture'. We speculate that the mechanism of this may relate to such 'holistic' GPs engaging in the 'life-world' of their patients rather than adhering solely to 'the voice of medicine' [18,19]. Direct observational studies on the actual consultations in deprived areas are required to test this hypothesis. Enhancing holistic care in deprived areas is of course a major challenge, given the gap that current exists regarding workforce capacity and population health needs [20], and the future effect on holism of the privatisation of primary care provision being rapidly introduced in deprived areas of England remains to be seen [21].

#### Doctor-patient relationship

The findings of the present study also relates to a number of other published qualitative studies on patients' views on the consultation and the doctor-patient relationship which have been carried out without a specific focus on socio-economic deprivation. The meaning of 'personal care' to patients and health care providers was recently reported [22], with human communication and individualised care emerging as the most important factors. The process of creating a relationship with a GP is an active, dynamic process [23] but the end result – a 'human relationship with a doctor' -has been found to means 'simple and obvious things' i.e., that patients want their doctor to take their symptoms seriously, to listen and/or ask questions about their symptoms, to treat them as a real person and not only a patient, and to ask questions about other things than the disease such as family or work issues [24].

#### Continuity of care

Several previous qualitative studies have also examined patients' views on continuity of care, a key 'structural' factor that emerged in the present study. A recent UK study found that seeing a known and trusted doctor was especially important to patients with chronic, complex, and emotional problems [25]. Other recent research in primary care in Europe has found that patients base seek interpersonal continuity of care with a GP in order to have sense of security based on four core foundations – (1) coherence (2) confidence in care (3) trusting relationship and (4) access [26]. Earlier studies confirm the importance of empathy, relationship, and a sense of partnership [27,28].

#### Consultation length

The limited time available in consultations was a second key 'structural ' factor identified in the present study, and was deeply inter-twined with patients' views on quality of consultation. Consultation length is shorter in the UK than in many other countries [29] and observational studies indicate that longer consultations are associated with better quality care [30,31]. Patients in deprived areas often receive shorter consultations than in more affluent areas despite higher levels of co-morbidity and psychological and physical health needs [12,31,32]. Although both GPs and patients would like more time in consultations [6,33], evidence of improvement in quality of care by actively providing longer consultations is limited [34]. Clearly this is an area which requires further research, as the provision of longer consultations has substantial implications for workload and resources.

### Meaning of study – explanation of implications for clinicians and policy makers

Health care in the UK is provided largely by the National Health Service (NHS), with a GP-led Primary care that deals with almost 90% of NHS activity. In Scotland, healthcare is a devolved issue, and the management structure of primary care differs somewhat. At the time the present study was carried out, primary care was locally organised in to 'Local Health Care Co-operative', small primary care organisations under the auspices of regional health boards. Recently, in an attempt to dovetail community care, social care, and primary care, LHCCs have been superseded by Community Health Care Partnerships (CHCPs) which are larger managerial units within the health board structure. This has little implications for the findings of the study, but may have implications for the way primary care can or cannot respond to patients' views on quality.

Doctors and patients consistently report empathy and humanness to be top priorities in their care [35-37]and the present study confirms the importance to patients of such values and skills in general practice in deprived areas. However recent UK and Scottish government policy initiatives have placed greater emphasis on rapid access to care and to patient-choice than on interpersonal, continuous care. A possible detrimental effect of the new GMS contract on such personal care has also recently been suggested [38].

Given the importance that patients attach to empathic care, it would seem appropriate to examine ways of measuring it in routine practice. Previous work by one of the authors (SWM) has led to the development and validation of the Consultation and Relational Empathy (CARE) Measure [39,40] which appears to capture the main themes of importance to patients in deprived and non-deprived areas. The CARE Measure is currently being used for GP Appraisal and for patient feedback of GPs in training, as part of workplace-based assessment under the auspices of the Post-Graduate Medical Education Training Board (PMETB) of the UK. It has been suggested that a combination of the CARE Measure with other measures of consultation quality may be useful in the evaluation and reward of GPs [41]. Recent research using these tools in general practice in deprived and affluent areas has shown links between empathy, patient enablement, time, and personal continuity of care [41]. Larger quantitative studies are required to delineate the importance of these factors in health outcomes and use of services in deprived and other areas, but the results of the present qualitative study would suggest that long-term commitment, genuine engagement and personal continuity of care, together with sufficient time in consultations, are pre-requisites for high quality holistic general practice in areas of high deprivation. However, in such areas of high deprivation, GPs report more stress [6,13] – with implications for burn-out and retention – and find it harder to provide the sort of personal care patients seek, due to the competing demands of access, co-morbidity, time, and workload. With the continuing existence of the inverse care law [12] – which the new GMS contract for GPs appears to be perpetuating [42] – it is hard to see how GPs in deprived areas can be expected to 'raise their game' further, without substantial redistribution of support and resource. However, recent the policy commitment to reduce inequalities in health through 'anticipatory care' in primary care in Scotland is an important step [10]. Evaluation of any new initiatives should, in our view, include measures of consultation quality from the patients' perspective [40,41].

### Unanswered questions and future research

Interestingly, there was very little explicit reference in the patients' narratives in the present study to 'shared decision making' – despite this being high on the research and policy agenda in the UK [44]. It may be that patients in deprived areas don't want or expect to share in decisions about their health care, or that they don't experience it and therefore it is not part of their expectations [43,44]. Further research is required on the issue of patient participation in deprived areas and relationship to outcomes, given the focus on self-care and self-management of chronic disease [45].

## Competing interests

The author(s) declare that they have no competing interests.

## Authors' contributions

SM will act as guarantor for the study. PC conceived and designed the study, helped collect data, and carried out an initial analysis and interpretation of the data. He assisted in the secondary analysis of the data. AB carried out the secondary analysis of the data and helped in interpretation of the data. She also helped revised the first draft. SM discussed the conception, helped in the secondary analysis and interpretation of the data, revised several versions of the manuscript, and also gave critical intellectual input into this process. All authors read and approved the final manuscript.

## Pre-publication history

The pre-publication history for this paper can be accessed here:


